# Morphology and Molecular Phylogeny of Two New Trachelophyllid Ciliates, *Monolamellophrya terricola* gen. nov., sp. nov. and *Trachelophyllum parapiculatum* sp. nov. (Litostomatea, Haptoria), From South Korea

**DOI:** 10.3389/fmicb.2022.893886

**Published:** 2022-06-06

**Authors:** Atef Omar, Ji Hye Moon, Jae-Ho Jung

**Affiliations:** ^1^Natural Science Research Institute, Gangneung-Wonju National University, Gangneung, South Korea; ^2^Department of Zoology, Al Azhar University, Assiut, Egypt; ^3^Department of Biology, Gangneung-Wonju National University, Gangneung, South Korea

**Keywords:** dorsal brush, Korea, lepidosomes, SSU rRNA, Trachelophyllidae

## Abstract

The morphology and molecular phylogeny of two new ciliates, *Monolamellophrya terricola* gen. nov., sp. nov. and *Trachelophyllum parapiculatum* sp. nov., discovered in South Korea, were investigated. The two species belong to the suborder Trachelophyllina, which is characterized by the presence of a mucilaginous layer containing lepidosomes covering the cortex. *Monolamellophrya terricola* gen. nov., sp. nov. is characterized by the presence of a single layer of type II lepidosomes, representing a new genus. *Trachelophyllum parapiculatum* sp. nov. has only type I lepidosomes covering the cortex, a generic character of the genus *Trachelophyllum*, and is distinguished from other congeners by a combination of morphological features, including the 15–24 μm long rod-shaped extrusomes, the 9–13 ciliary rows, the 7–11 and 17–25 dikinetids in brush rows 1 and 2, respectively, and the bipolar brush row 3. Furthermore, the 18S rRNA gene sequences of the two new species were provided. The phylogenetic analyses show that the sequence of *M. terricola* gen. nov., sp. nov. clusters with two other trachelophyllid sequences, and the sequence of *T. parapiculatum* sp. nov. is placed at the base of these three sequences with full support. Furthermore, the four trachelophyllid sequences that are available so far form a monophyletic clade.

## Introduction

Haptorid ciliates of the suborder Trachelophyllina Grain, 1994 are widely distributed and mostly found in terrestrial and semiterrestrial habitats but also found in freshwater and rarely in saline water (Kahl, 1930; Foissner, 1984, 1994, 2005, 2016; Foissner et al., 1995, 1999, 2002; Coats and Clamp, 2009; Telesh et al., 2009; Jang et al., 2015; Bourland, 2017). They are characterized by the presence of organic epicortical scales, of unknown function called lepidosomes, embedded in a mucilaginous layer covering the cortex. The shape of the lepidosomes is considered a genus-specific character within the Trachelophyllina and can only be revealed using a scanning electron microscope, thus identification of these taxa based only on live observation and/or silver impregnation is insufficient (Foissner et al., 2002; Foissner, 2005, 2016). So far, 12 types of lepidosomes were described in 19 well-characterized species and ten genera. Each genus is characterized by the presence of one, two, or three types of lepidosomes as follows: the genera *Epitholiolus* Foissner et al., 2002 and *Trachelophyllum* Claparéde and Lachmann, 1859 each has only a single type of lepidosomes; the genera *Bilamellophrya* Foissner et al., 2002, *Cataphractes* Foissner, 2016, *Ileonema* Stokes, 1884, *Lingulothrix* Foissner et al., 2002, *Sleighophrys* Foissner, 2005, and *Spetazoon* Foissner, 1994 each has two types of lepidosomes; and the genera *Luporinophrys* Foissner, 2005 and *Trachelophyllides* Foissner, 2016 each has three types of lepidosomes (Nicholls and Lynn, 1984; Foissner et al., 2002; Foissner, 2005, 2016; Bourland, 2017). Trachelophyllina genera belong to two different families mainly based on the dorsal brush rows organization. The family Lingulotrichidae Foissner, 2016 is characterized by the presence of more than three heteromorphic brush rows, while the family Trachelophyllidae Kent, 1881 is characterized by the presence of two dikinetidal and one monokinetidal brush rows (Foissner, 2016). Currently, the trachelophyllids are highly underrepresented in the phylogenetic trees, with only two 18S rRNA gene sequences available in the GenBank database. Unfortunately, the identification of these two sequences is doubtful because data on their lepidosomes are lacking (Vdačný et al., 2011; Jang et al., 2015, 2022; Huang et al., 2018).

In this study, we investigate the morphology of two species discovered in South Korea. The first species is unique within the Trachelophyllidae in having a single layer of type II lepidosomes and thus representing a new genus, *Monolamellophrya* gen. nov., while the second species has only type I lepidosomes as other members of the genus *Trachelophyllum* and is distinguishable from other congeners by a combination of features, i.e., the 15–24 μm long rod-shaped extrusomes, the 9–13 ciliary rows, the 7–11 and 17–25 dikinetids in brush rows 1 and 2, respectively, and the bipolar brush row 3. Moreover, the 18S rRNA gene sequences of the two species were analyzed to determine their phylogenetic position.

## Materials and Methods

### Sample Collection and Identification

*Monolamellophrya terricola* gen. nov., sp. nov. was discovered in a soil sample collected near Bongnae Falls, Ulleung Island, South Korea (N 37° 29′ 48.12″ E 130° 53′ 29.64″) in August 2018. The soil sample was air-dried for at least 2 weeks and stored in a plastic bag. The sample was rewetted in February 2019 with mineral water (Jeju Samdasoo, Jeju Province Development Co., South Korea) to induce excystment of ciliates using the non-flooded Petri dish method (Foissner et al., 2002). *Trachelophyllum parapiculatum* sp. nov. was discovered in a water sample including debris collected from a temporary pond on a lawn in the Gangneung-Wonju National University, Gangneung-si, South Korea (N 37°46′12.4″ E 128°52′16.5″) after heavy rainfall in July 2020. The water sample was kept in a plant culture dish at room temperature. Attempts to establish enriched cultures were unsuccessful for both species. Living specimens were investigated using a stereomicroscope (Olympus SZ61, Tokyo, Japan) and a light microscope (Olympus BX53) with differential interference contrast at magnifications of 50–1,000×. The infraciliature was revealed by protargol impregnation and scanning electron microscopy. Protargol powder was synthesized using the methods described by Pan et al. (2013) and Kim and Jung (2017), and the protargol impregnation technique is based on ‘procedure A' described by Foissner (2014). The SEM technique was conducted following the procedures described by Foissner (2014) and Moon et al. (2020). The terminology is according to Foissner et al. (2002) and Foissner (2016).

### DNA Extraction, PCR Amplification, and Sequencing

Under the stereomicroscope, five cells were collected using a microcapillary from raw cultures of both species. The cells were transferred to habitat water filtered by a 0.2-μm syringe filter (Minisart® CA Syringe Filters; Sartorius, Aubagne, France), starved for at least 3 h, washed at least five times using the same filtered water to remove other eukaryotes, and then transferred to a 1.5-ml centrifuge tube each with a minimum volume of water. Genomic DNA was extracted using a RED-Extract-N-Amp Tissue PCR Kit (Sigma, St. Louis, MO, USA). The 18S rRNA gene was amplified using the primer New Euk A (5′-CTG GTT GAT YCT GCC AGT-3′) (Moon et al., 2017), which is a slightly modified version of the primer Euk A in Medlin et al. (1988), and the primer LSU rev4 (5′-GTT AGA CTY CTT GGT CCG TG-3′) (Sonnenberg et al., 2007) for *M. terricola* gen. nov., sp. nov. The primers New Euk A and Euk B (5′-TGA TCC TTC TGC AGG TTC ACC TAC-3′) were used to cover nearly the entire 18S rRNA gene of *T. parapiculatum* sp. nov. The PCR conditions for the primers New Euk A and LSU rev4 were as follows: denaturation at 94°C for 1 min 30 s, followed by 40 cycles of denaturation at 98°C for 10 s, annealing at 58.5°C for 30 s, and extension at 72°C for 3 min, and a final extension step at 72°C for 7 min. The PCR conditions for the primers New Euk A and Euk B were as follows: denaturation at 94°C for 90 s, followed by 40 cycles of denaturation at 98°C for 10 s, annealing at 58.5°C for 30 s, extension at 72°C for 2 min, and a final extension step at 72°C for 7 min. For purification of the PCR products, MEGAquickspin Total Fragment DNA Purification Kit (iNtRON Biotechnology, South Korea) was used. DNA sequencing was performed using an ABI 3700 sequencer (Applied Biosystems, Foster City, CA, USA). New Euk A, LSU rev4, and three internal primers [18SF790v2: 5′-AAA TTA KAG TGT TYM ARG CAG-3′, 18SR300: 5′-CAT GGT AGT CCA ATA CAC TAC-3′ (Park et al., 2017), and 18SF1470: 5′-TCT GTG ATG CCC TTA GAT GTC-3′ (Jung et al., 2018)] were used for the sequences of *M. terricola* gen. nov., sp. nov., and only New Euk A and Euk B primers were used for the sequences of *T. parapiculatum* sp. nov.

### Phylogenetic Analyses

The 18S rRNA gene sequences of *M. terricola* gen. nov., sp. nov. and *T. parapiculatum* sp. nov. were assembled using Geneious 9.1.5 (Kearse et al., 2012). To determine the phylogenetic position of the two new species, 18S rRNA gene sequences of 77 ciliates were retrieved from the NCBI database, including three metopids as outgroup taxa: *Clevelandella panesthiae* (KC139719), *Metopus palaeformis* (AY007450), and *Nyctotherus ovalis* (AJ222678). The sequences were aligned using ClustalW (Thompson et al., 1994), and both ends were manually trimmed using BioEdit 7.0.9.0 (Hall, 1999). The length of the final alignment was 1609 bp. Using jModelTest 2.1.7 (Darriba et al., 2012), the best-fit evolutionary model TVM+I+G under the Akaike information criterion (AIC) was selected. The maximum likelihood (ML) tree was constructed using IQ-Tree 1.5.3 (Nguyen et al., 2015). The reliability of internal branches was assessed using a nonparametric bootstrap method with 100,000 replicates. The number of nucleotide differences and pairwise sequence similarity was calculated using MEGA 6.06 (Tamura et al., 2013). MrBayes 3.1.2 (Ronquist et al., 2012) was used for Bayesian inference (BI) analyses with Markov Chain Monte Carlo for 3,000,000 generations at a sampling frequency of every 100 generations, and the first 25% of trees were discarded as burn-in. Phylogenetic trees were visualized using the free software package FigTree version 1.4.3 by Rambaut (2006).

## Results

ZooBank registration number of this study: urn:lsid:zoobank.org:pub:CE631A43-5895-4080-95EF-D43022C95A78

ZooBank registration number of *Monolamellophrya* gen. nov.: urn:lsid:zoobank.org:act:74B5B8AA-E968-47A7-AF9A-6F233605EE36

ZooBank registration number of *M. terricola* gen. nov., sp. nov.: urn:lsid:zoobank.org:act:2BAE8213-FC5D-4DD5-9472-03C79245E819

ZooBank registration number of *T. parapiculatum* sp. nov.: urn:lsid:zoobank.org:act:A49DCDEF-7A93-477D-AEC5-F6CAEBADF046

### Taxonomy

Phylum Ciliophora Doflein, 1901

Subphylum Intramacronucleata Lynn, 1996

Class Litostomatea Small and Lynn, 1981

Subclass Haptoria Corliss, 1974

Order Spathidiida Foissner and Foissner, 1988

Suborder Trachelophyllina Grain, 1994

Family Trachelophyllidae Kent, 1881

### *Monolamellophrya* gen. nov.

#### Diagnosis

Trachelophyllidae with only one layer of type II lepidosomes. Lepidosomes with conical superstructure composed of six or seven concave meridional arcs.

#### Etymology

Composite of the Greek numeral *mono* (one), the Latin noun *lamella* (thin plate), and the Greek noun *ophrya* (eyebrow, cilia, ciliate), referring to the single type of lepidosome. Feminine gender.

#### Type Species

*Monolamellophrya terricola* sp. nov.

#### Species Assignable

Only the type species.

### *Monolamellophrya terricola* gen. nov., sp. nov.

#### Diagnosis

Size *in vivo* 100–160 × 15–25 μm; body slenderly fusiform and slightly flattened dorsoventrally. Two macronuclear nodules and two micronuclei. Extrusomes acicular, 7–9 μm long in oral bulge and cytoplasm. 11–13 meridional ciliary rows. Dorsal brush rows 1 and 2 dikinetidal and isostichad, each consisting of 11–18 and 12–18 dikinetids, respectively; brush row 3 monokinetidal, slightly longer than rows 1 and 2. Lepidosomes of type II, ~ 1.0 × 0.7 × 0.6 μm, with conical superstructure composed of six or seven arcs.

#### Etymology

The Latin species-group name *terricola* (living in soil) refers to the habitat in which the species was discovered.

#### Type Locality

Soil near Bongnae Falls, Ulleung Island, South Korea (N 37° 29′ 48.12″ E 130° 53′ 29.64″).

#### Type Material

**Figure 1:**
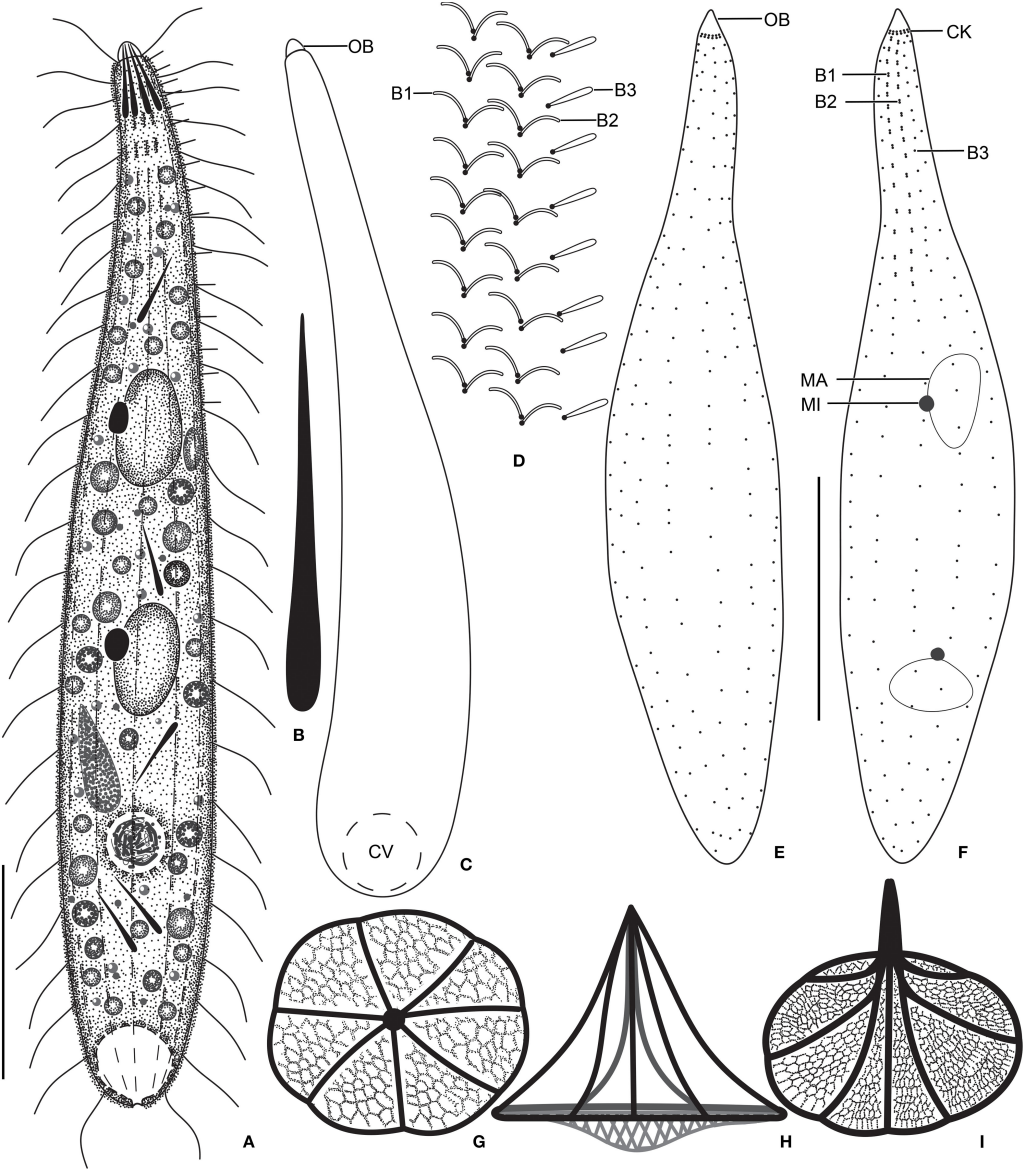
*Monolamellophrya terricola* gen. nov., sp. nov. from life **(A–D)**, after protargol impregnation **(E,F)**, and redrawn from scanning electron micrographs **(G–I)**. **(A)** A representative specimen, showing the body shape and the acicular extrusomes in the oral region and cytoplasm. **(B)** Acicular extrusome. **(C)** A specimen with broadly rounded posterior end. **(D)** Ciliature of dorsal brush. **(E,F)** Ventral **(E)** and dorsal **(F)** views of the holotype specimen, showing the infraciliature and the nuclear apparatus. **(G–I)** Vertical **(G)**, lateral **(H)**, and oblique distal **(I)** views of the type II lepidosomes, showing the circular baseplate with convex middle portion, the fine arcs forming rosette-like structure in surface view **(G)**, and a conspicuous cone structure in lateral view **(H)**. B1–3, dorsal brush rows; CK, circumoral kinety; CV, contractile vacuole; MA, macronucleus; MI, micronucleus. Scale bars = 30 μm.

The slide containing the holotype (Figures 1E,F, **3A**; NNIBRPR21237) and one paratype slide (NNIBRPR21238) with protargol-impregnated specimens were deposited at the Nakdonggang National Institute of Biological Resources (NNIBR), Sangju, Korea.

### Morphological Description of *Monolamellophrya terricola* gen. nov., sp. nov.

**Figure 2:**
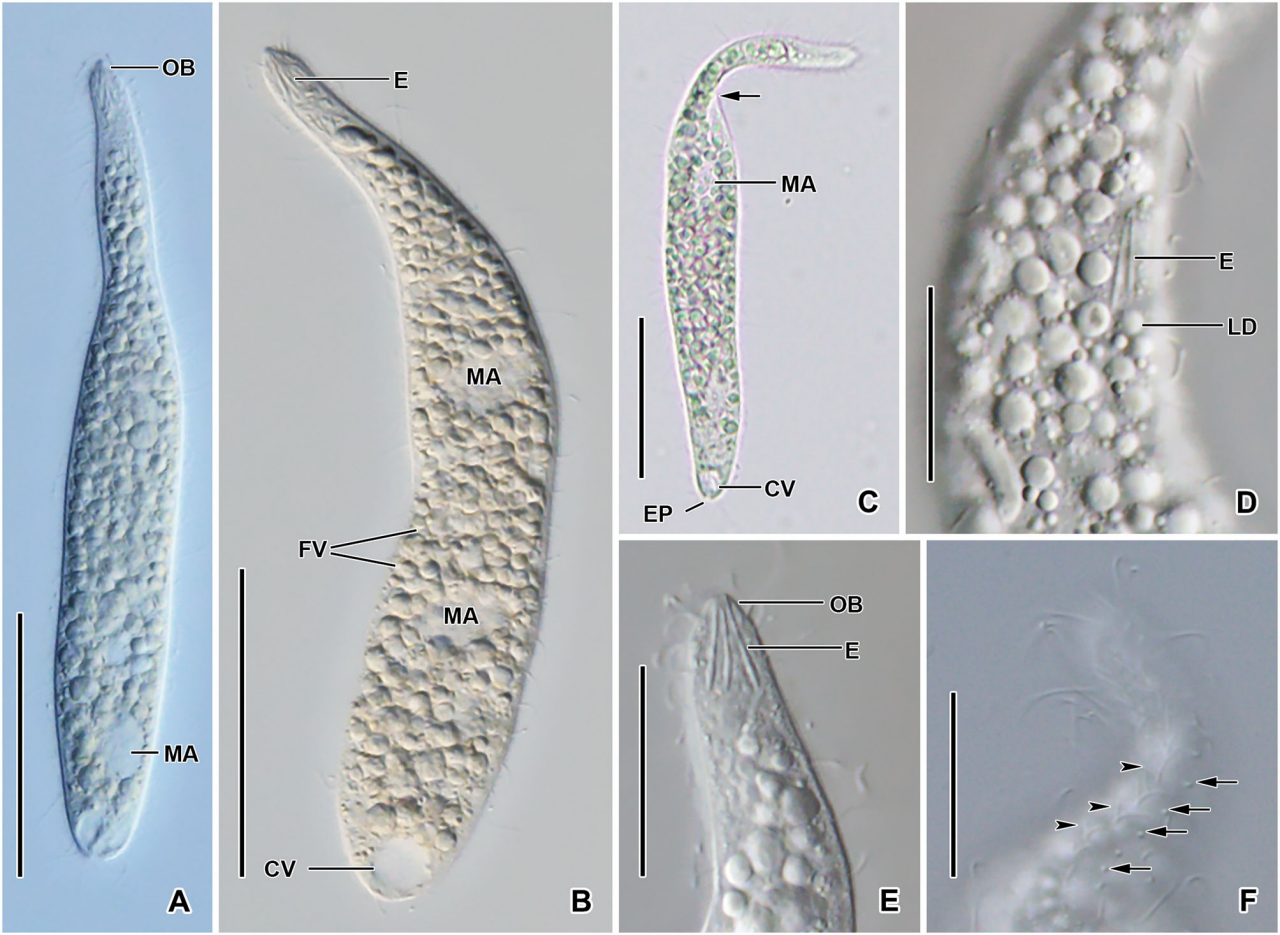
*Monolamellophrya terricola* gen. nov., sp. nov. from life. **(A–C)** Overviews showing the body shape, the narrow neck, the terminal contractile vacuole, the nuclear apparatus, and the cytoplasm studded with food vacuoles and lipid droplets. The arrow shows that the body is laterally flattened. **(D,E)** Optical sections showing the acicular extrusomes in cytoplasm **(D)** and oral bulge **(E)** and the lipid droplets. **(F)** The dorsal brush, showing the curved, V-like spread bristles of brush row 2 (arrowheads) and the acicular bristles of brush row 3 (arrows). B1–3, dorsal brush rows; CK, circumoral kinety; CV, contractile vacuole; E, extrusomes; EP, excretory pore; FV, food vacuoles; LD, lipid droplets; MA, macronucleus; MI, micronucleus; OB, oral bulge. Scale bars = 50 μm **(A–C)** and 20 μm **(D–F)**.

**Figure 3:**
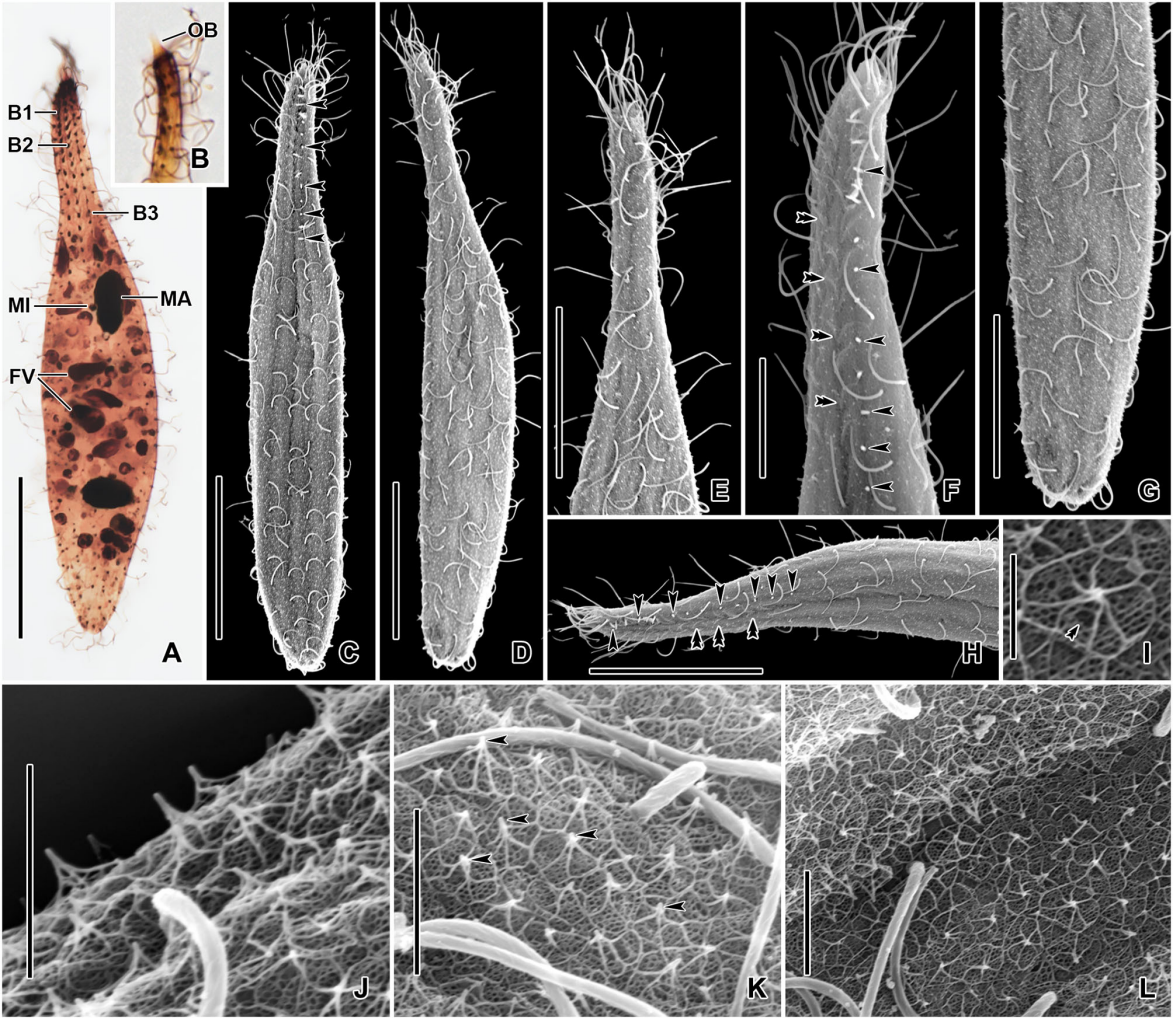
*Monolamellophrya terricola* gen. nov., sp. nov. after protargol impregnation **(A,B)** and in the scanning electron microscope **(C–L)**. **(A)** Dorsal view of the holotype specimen showing the dorsal brush rows, the nuclear apparatus, and the cytoplasm packed with food vacuoles. **(B)** The narrow, conical oral bulge of a paratype specimen. **(C–H)** Dorsal **(C,F,H)** and ventral **(D,E,G)** views, showing the cortex covered with epicortical scales of type II indicated by the white dots, which represent the cone structure made by the fine arcs. Arrowheads mark the bristles of the dorsal brush row 3. Cilia of brush rows 1 and 2 V-like spread and covered by epicortical scales (double arrowheads). **(I–L)** Lepidosomes of type II showing the fine arcs fusing together and forming a rosette-like structure in the top view. The double arrowhead in **(I)** marks the depression in the middle area of the baseplate. B1–3, dorsal brush rows; FV, food vacuoles; MA, macronucleus; MI, micronucleus. Scale bars = 30 μm **(A,C,D,H)**, 20 μm **(E,G)**, 10 μm **(F)**, 2 μm **(J,K)**, and 1 μm **(I)**.

Size 100–160 × 15–25 μm *in vivo* and 75–137 × 11–23 μm after protargol impregnation; length:width ratio ~ 6.7:1 *in vivo* and 3.9–11.4:1 (6.1:1 on average) after protargol impregnation. Body slenderly fusiform, slightly flattened dorsoventrally, with slightly (in contracted specimens) to distinctly (in extended specimens) narrow neck, ~ 5 μm wide in protargol preparations, slightly widened in the oral region, and gradually broadened posteriorly merging into the wider, slender trunk. The anterior end (oral bulge) narrow and conical, the posterior end usually narrowly rounded, rarely broadly rounded (Figures 1A,C,E,F, 2A–C, 3A–H). Cells very flexible and contractile by up to 30% of body length under cover glass; contracts and extends very slowly. The nuclear apparatus usually in the middle third of the cell, sometimes posterior macronuclear nodule displaced to a posterior quarter of the cell. Macronuclear nodules usually ellipsoidal to narrowly ellipsoidal and sometimes with irregular outline, each ~ 15 × 7 μm *in vivo*. Micronuclei near or attached to macronuclear nodules, globular to broadly ellipsoidal (Figures 1A,F, 2A–C, 3A). Contractile vacuole in the posterior body end with a single terminal excretory pore (Figures 1A,C, 2A–C). Extrusomes acicular, 7–9 μm long *in vivo*, form bundle in the oral bulge and scattered in the cytoplasm, and do not impregnate with protargol (Figures 1A,B, 2D,E). Cytoplasm colorless, contains lipid droplets 1–4 μm across and food vacuoles up to 10 μm across containing flagellates and small ciliates (Figures 1A, 2A–E, 3A–E). Usually glides slowly between soil particles and occasionally swims slowly by rotating about the main body axis.

Cortex thin and very flexible, covered with a very thin mucilaginous layer of lepidosomes, recognizable only in scanning electron micrographs. Mucilaginous layer composed of a single layer of tightly spaced type II lepidosomes (Figures 3C–L). Individual lepidosomes 1.0 × 0.7 × 0.6 μm on average in scanning electron micrographs, baseplate circular to broadly ellipsoidal, finely faceted, central area slightly to distinctly convex (Figures 1H, 3I), gives rise to six or seven fine, concave arcs, forming cone-shaped superstructure in lateral view and a rosette-like structure in top view (Figures 1G–I, 3I–L).

Cilia *in vivo* 8–10 μm in length. 11–13 meridional and equidistant ciliary rows composed of rather widely spaced monokinetids, three of them form dorsal brush rows anteriorly and continue posteriorly as ordinary somatic ciliary rows (Figures 1D–F, 3A–H). Brush rows 1 and 2 of similar length and structure (isostichad), extend to ~36% of body length and composed of rather widely spaced 11–18 and 12–18 dikinetids, respectively, dikinetids bear curved, immobile, V-like spread bristles, 3–4 μm long; bristles laying down and covered by mucilaginous layer and hardly recognizable both *in vivo* and in scanning electron micrographs (Figures 1D,F, 2F, 3H). Brush row 3 slightly longer than rows 1 and 2, composed of widely spaced monokinetids bearing 2–3 μm long, acicular and immobile bristles, piercing scale layer and recognizable both *in vivo* and in scanning electron micrographs (Figures 1D,E, 2F, 3A–C,F,H; Table 1).

**Table 1:** Morphometric data on *Monolamellophrya terricola* gen. nov., sp. nov. (Mt), and *Trachylophyllum parapiculatum* sp. nov. (Tp).

**Characteristic^**a**^**	**Species**	**Mean**	**Median**	**SD**	**SE**	**CV**	**Min**	**Max**	***n***
Body, length	Mt	97.6	98.0	14.2	3.7	14.5	75.0	137.0	15
	Tp	119.4	115.0	12.2	3.3	10.2	107.0	150.0	14
Body, width	Mt	17.0	17.0	3.7	0.9	21.6	11.0	23.0	15
	Tp	15.1	14.5	3.5	0.9	22.9	9.0	20.0	14
Body length:width, ratio	Mt	6.1	5.4	2.0	0.5	32.4	3.9	11.4	15
	Tp	8.4	7.6	2.9	0.8	34.0	5.6	16.7	14
Oral bulge, height	Mt	2.0	2.0	0.3	0.1	14.6	1.5	3.0	15
	Tp	3.6	4.0	0.7	0.2	18.0	3.0	5.0	13
Oral bulge, diameter	Mt	1.9	2.0	0.4	0.1	20.4	1.5	3.0	15
	Tp	4.5	5.0	0.7	0.2	14.5	3.0	5.0	13
Circumoral kinety to last dikinetid of brush row 1, distance	Mt	32.7	32.0	6.0	1.5	18.3	25.0	50.0	15
	Tp	12.4	12.0	1.6	0.4	12.6	10.0	15.0	13
Brush row 1, number of dikinetids	Mt	13.9	13.0	2.1	0.6	15.4	11.0	18.0	15
	Tp	8.9	9.0	1.2	0.4	13.9	7.0	11.0	12
Circumoral kinety to last dikinetid of brush row 2, distance	Mt	30.1	30.0	5.8	1.5	19.2	21.0	46.0	15
	Tp	26.1	25.0	4.1	1.1	15.7	21.0	35.0	13
Brush row 2, number of dikinetids	Mt	14.2	13.0	2.0	0.5	13.9	12.0	18.0	15
	Tp	20.3	20.0	2.3	0.6	11.1	17.0	25.0	13
Brush row 3, number of monokinetids	Mt	16.7	16.0	1.5	0.4	9.3	14.0	19.0	15
	Tp	34.3	33.0	3.3	0.9	9.5	30.0	41.0	12
Anterior body end to anterior macronucleus, distance	Mt	41.1	38.0	10.6	2.7	25.8	30.0	70.0	15
	Tp	42.7	41.0	7.4	2.0	17.4	29.0	54.0	14
Anterior macronuclear nodule, length	Mt	11.6	12.0	2.3	0.6	19.5	7.0	15.0	15
	Tp	10.9	10.5	2.9	0.8	26.3	6.0	15.0	14
Anterior macronuclear nodule, width	Mt	5.6	5.0	1.0	0.3	17.6	4.0	8.0	15
	Tp	5.6	5.5	1.3	0.4	24.1	4.0	9.0	14
Anterior macronucleus nodule length:width, ratio	Mt	2.2	2.2	0.7	0.2	31.9	1.0	3.8	15
	Tp	2.1	1.8	0.9	0.2	41.9	0.9	3.8	14
Macronuclear nodules, number	Mt	2.0	2.0	0.0	0.0	0.0	2.0	2.0	15
	Tp	2.0	2.0	0.0	0.0	0.0	2.0	2.0	14
Micronucleus, length	Mt	2.7	2.5	0.7	0.2	28.1	2.0	4.0	13
	Tp	2.2	2.0	0.6	0.2	26.6	2.0	4.0	12
Micronucleus, width	Mt	2.0	2.0	0.2	0.1	11.8	1.5	2.5	10
	Tp	2.2	2.0	0.6	0.2	26.6	2.0	4.0	12
Micronucleus length:width, ratio	Mt	1.3	1.0	0.4	0.1	32.4	1.0	2.0	10
	Tp	1.0	1.0	0.0	0.0	0.0	1.0	1.0	12
Micronuclei, number	Mt	2.0	2.0	0.0	0.0	0.0	2.0	2.0	10
	Tp	1.8	2.0	0.4	0.1	20.3	1.0	2.0	13
Nuclear figure, length	Mt	36.9	34.0	7.6	2.0	20.7	22.0	54.0	15
	Tp	39.8	40.0	8.7	2.4	21.8	20.0	54.0	13
Macronuclear nodules, distance in between	Mt	15.9	16.0	7.3	1.9	45.9	4.0	32.0	15
	Tp	17.7	18.0	6.0	1.6	34.0	7.0	26.0	14
Ciliary rows, number	Mt	11.5	11.0	0.7	0.2	6.5	11.0	13.0	15
	Tp	11.2	11.0	1.0	0.3	8.7	9.0	13.0	14
Kinetids in a ventral kinety, number	Mt	27.9	27.0	4.1	1.1	14.7	23.0	38.0	15
	Tp	29.6	29.0	3.9	1.1	13.2	24.0	38.0	13
Dikinetidal brush rows, number	Mt	2.0	2.0	0.0	0.0	0.0	2.0	2.0	15
	Tp	2.0	2.0	0.0	0.0	0.0	2.0	2.0	14

a *Data based on protargol-impregnated specimens. Measurements in μm. CV, coefficient of variation in %; M, median; Max, maximum; Mean, arithmetic mean; Min, minimum; n, number of individuals investigated; SD, standard deviation; SE, standard error of arithmetic mean*.

Oral bulge inconspicuous *in vivo* and after protargol impregnation because of hyaline and small size (2 × 2 μm in stained cells); conical and set off from body proper, contains extrusomes. Circumoral kinety at the base of the oral bulge, composed of ~12 dikinetids, each bearing a single cilium and inconspicuous nematodesmata extending posteriorly forming a pharyngeal basket (Figures 1A,C,E,F, 2A,B,E).

### *Trachelophyllum parapiculatum* sp. nov.

#### Diagnosis

Size *in vivo* 140–200 × 15–25 μm; body slenderly clavate to fusiform and dorsoventrally flattened. Two macronuclear nodules and two micronuclei. Extrusomes rod-shaped, 15–24 μm in length, forming a bundle in the oral bulge and scattered or forming bundles in the cytoplasm. 9–13 meridional ciliary rows. Brush rows distinctly heterostichad, rows 1 and 2, each consists of 7–11 and 17–25 dikinetids, respectively, row 1 approximately half length of row 2; brush row 3 monokinetidal, extends to posterior body end. Lepidosomes of type I, ~1.4 × 1.1 × 0.4 μm, with hemispherical superstructure composed of ~11 polygons.

#### Etymology

Composite of the Greek prefix *para-* (besides, like, resembling) and the species-group name *apiculatum*, referring to the similarity to *T. apiculatum* (Perty, 1852) Claparéde and Lachmann, 1859.

#### Type Locality

Temporary pond on a lawn in the Gangneung-Wonju National University, Gangneung, South Korea (N 37°46′12.4″ E 128°52′16.5″).

#### Type Material

**Figure 4:**
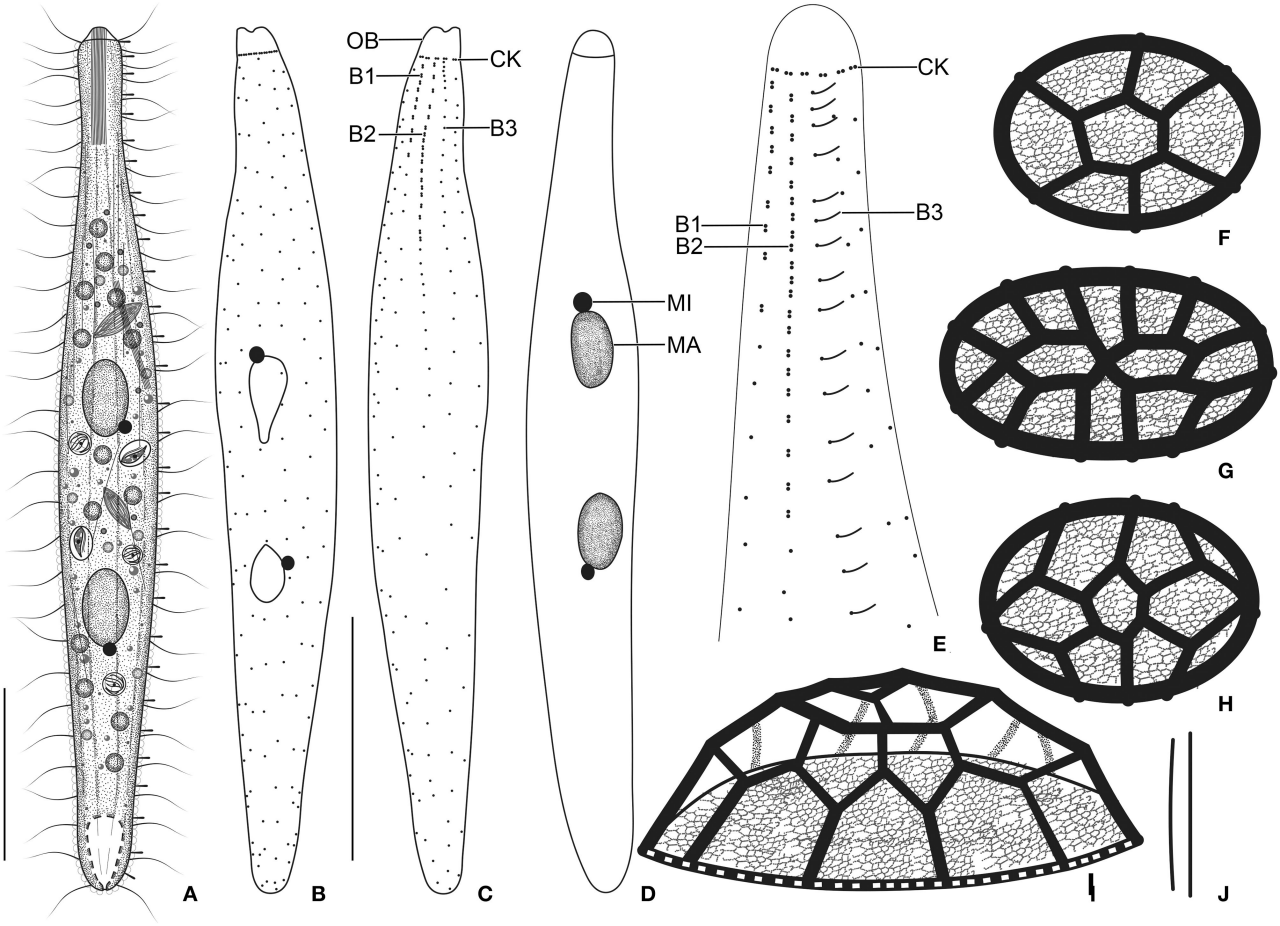
*Trachelophyllum parapiculatum* sp. nov. from life **(A,J)**, after protargol impregnation **(B–E)**, and redrawn from scanning electron micrographs **(F–I)**. **(A)** A representative specimen showing the body shape and the long extrusomes in the oral bulge and cytoplasm, and the long brush row 3. **(B–E)** Ventral **(B,D)** and dorsal **(C,E)** views of the holotype **(B,C)** and two paratype **(D,E)** specimens, showing the somatic ciliature and the nuclear apparatus. Note the two dikinetidal brush rows, the single monokinetidal brush 3, and the dikinetidal circumoral kinety. **(F–I)** Surface **(F–H)** and lateral **(I)** views of lepidosomes type I. **(J)** Extrusomes. B1–3, dorsal brush rows; CK, circumoral kinety; MA, macronuclear nodules; MI, micronucleus; OB, oral bulge. Scale bars = 30 μm.

The slide containing the holotype (Figures 4B,C, 6A,B; NNIBRPR21239) and one paratype slide (NNIBRPR21240) with protargol-impregnated specimens were deposited at the Nakdonggang National Institute of Biological Resources (NNIBR), Sangju, Korea.

### Morphological Description of *Trachelophyllum parapiculatum* sp. nov.

**Figure 5:**
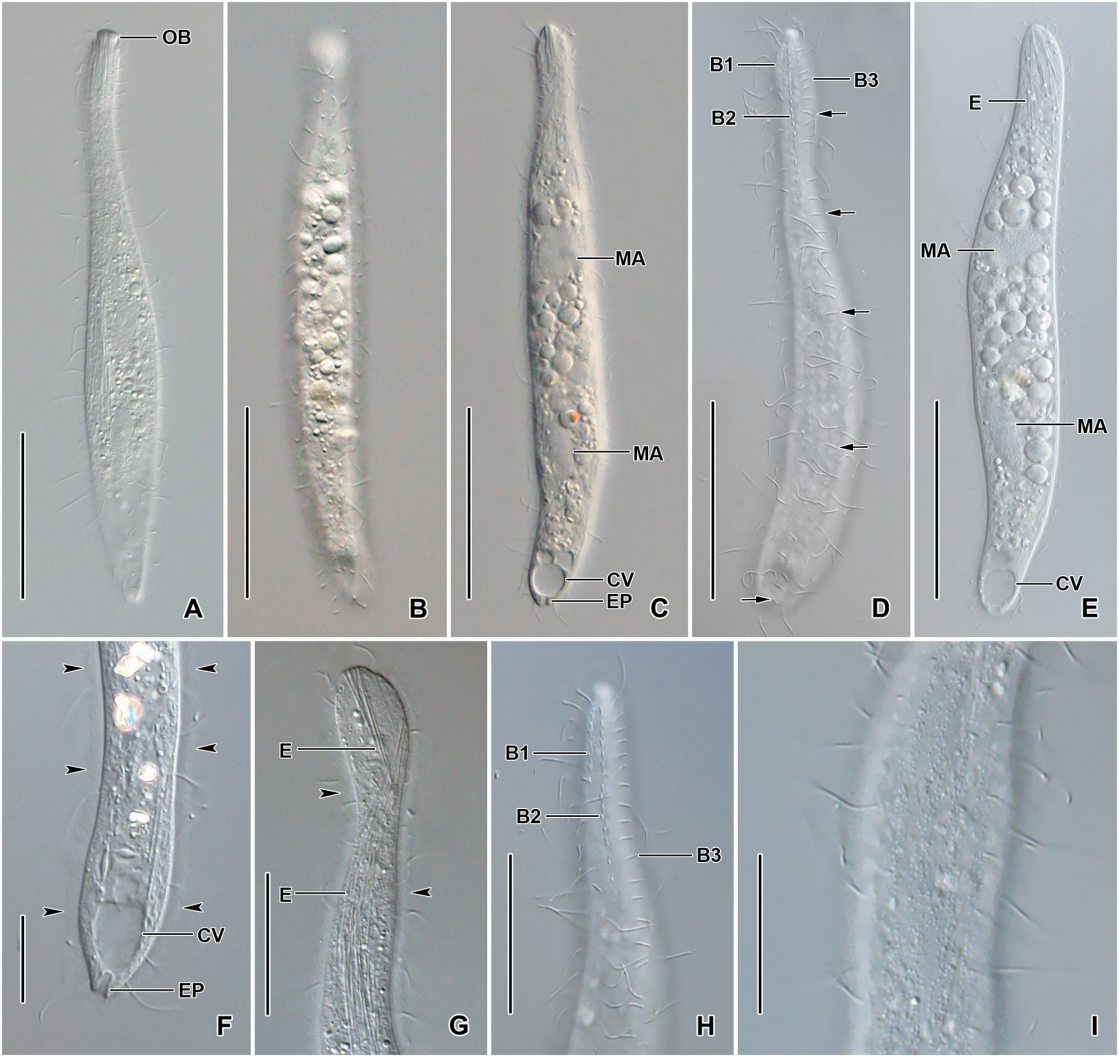
*Trachelophyllum parapiculatum* sp. nov. from life. **(A–E)** Overviews showing the body shape, the two widely spaced macronuclear nodules, the terminal contractile vacuole, the excretory pore, and the long rod-shaped extrusomes. Arrows denote the bipolar dorsal brush row 3. **(F,G)** Posterior **(F)** and anterior **(G)** body portion showing the contractile vacuole, the single excretory pore, and the extrusomes extending into the oral bulge. Arrowheads mark the mucilaginous layer. **(H)** Dorsal view of the anterior body portion showing the two dikinetidal and one monokinetidal brush rows. **(I)** Surface view showing the cortical granulation. B1–3, dorsal brush rows; CV, contractile vacuoles; E, extrusomes; EP, excretory pore; MA, macronuclear nodules; OB, oral bulge. Scale bars = 50 μm **(A–E)**, 30 μm **(H)**, 20 μm **(G,I)**, and 15 μm **(F)**.

**Figure 6:**
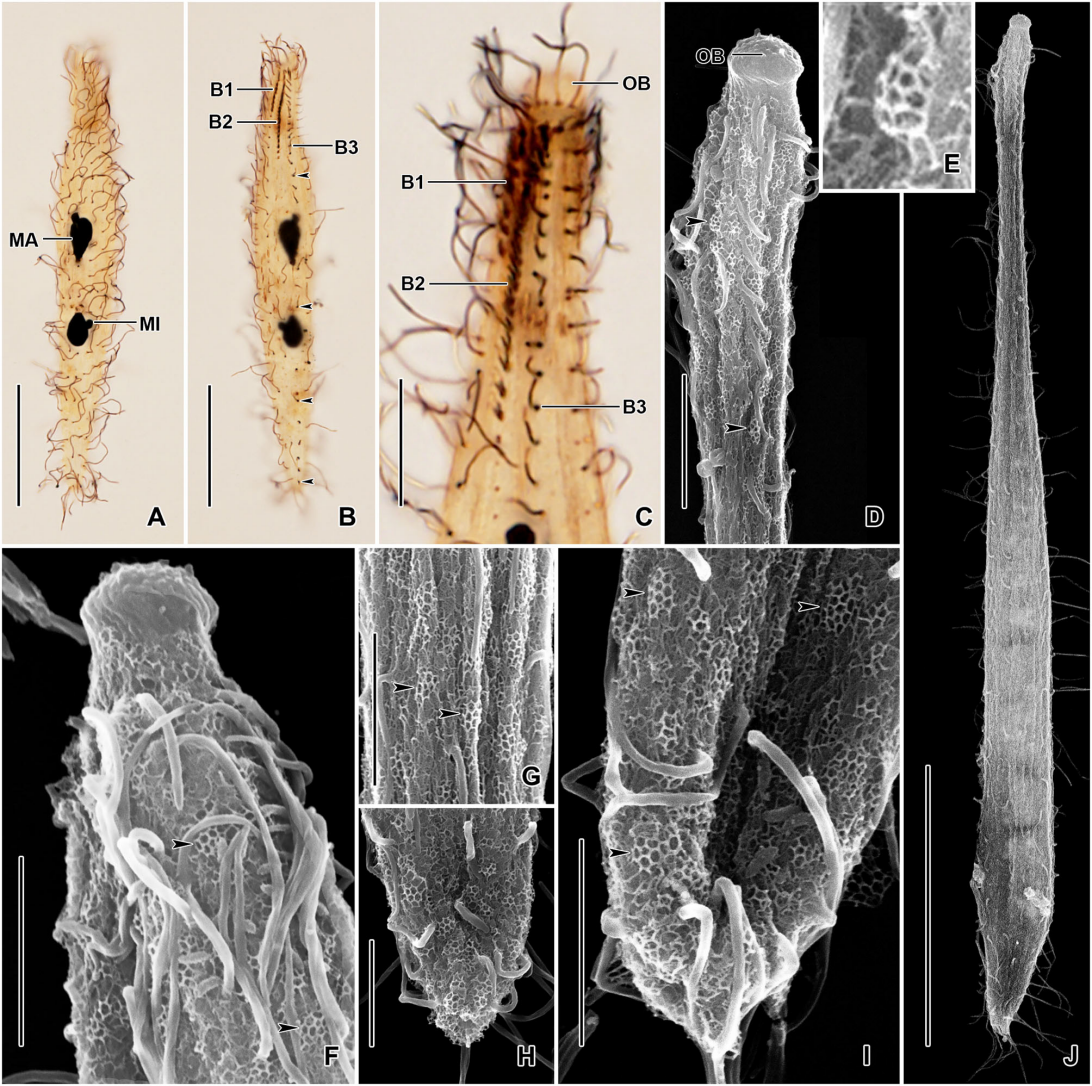
*Trachelophyllum parapiculatum* sp. nov. after protargol impregnation **(A–C)** and in the scanning electron microscope **(D–I)**. **(A–C)** Ventral **(A)** and dorsal **(B,C)** views of the holotype **(A,B)** and a paratype **(C)** specimen, showing the somatic ciliature, the dikinetidal dorsal brush rows 1 and 2, and the monokinetidal (arrowheads) brush row 3. Note the circumoral dikinetids each bearing a single cilium **(C)**. **(D,F)** Anterior body portion, showing the oral bulge not covered with lepidosomes. Arrowheads show type I lepidosomes. **(E)** Lateral view of lepidosome type I. **(G–I)** Cortex covered with type I lepidosomes (arrowheads). **(J)** An extended specimen. B1–3, dorsal brush rows; MA, macronuclear nodules; MI, micronucleus; OB, oral bulge. Scale bars = 50 μm **(J)**, 30 μm **(A,B)**, 10 μm **(C,D)**, and 5 μm **(F–I)**.

Size 140–200 × 15–25 μm *in vivo* and 107–150 × 9–20 μm after protargol impregnation; length:width ratio ~6.7:1 *in vivo* and 5.6–16.7:1 (on average 8.4:1) after protargol impregnation; body dorsoventrally flattened up to 2:1. Body slenderly clavate to fusiform with the neck slightly widened in the oral region and gradually broadened posteriorly merging into a wider trunk. The anterior end (oral bulge) narrow and cylindrical, posterior end narrowly rounded (Figures 4A–D, 5A–E,G,H, 6A,B,I). Cells very flexible and slightly contractile under cover glass; contracts and extends very slowly. The nuclear apparatus usually in the middle quarters of the cell. Macronuclear nodules usually ellipsoidal to narrowly ellipsoidal, each ~ 15 × 8 μm *in vivo*. Micronuclei near or attached to macronuclear nodules, spherical (Figures 4A,B,D, 5C,E, 6A,B). Contractile vacuole in posterior body end and connects with single terminal excretory pore by a distinct excretory canal (Figures 4A, 5C,E,F). Extrusomes straight or slightly curved rod-shaped, 15–24 μm long *in vivo*, form bundles in the oral region and extend into the oral bulge and scattered or form bundles in the cytoplasm, do not impregnate with protargol (Figures 4A,J, 5A,E,G). Cytoplasm colorless, contains 1–3 μm across lipid droplets and up to 10 μm across food vacuoles containing flagellates and small ciliates (Figures 4A, 5B,C,E). Usually swims slowly and rarely glides on the bottom of the culture dish.

Cortex thin and very flexible, contains small and irregularly arranged cortical granules (Figure 5I), covered by a mucilaginous layer of lepidosomes, recognizable *in vivo* and scanning electron micrographs. Mucilaginous layer composed of tightly spaced type I lepidosomes (Figures 4A, 5F,G, 6D–H). Individual scales 1.0–2.1 × 0.7–1.7 × 0.3–0.5 μm (1.4 × 1.1 × 0.4 μm on average; n = 12) in scanning electron microscopes, baseplate circular to elliptical finely faceted, perforations in baseplate margin hardly recognizable, superstructure hemispherical composed of an average of 11 polygons (Figures 4F–I, 6D–H).

Cilia *in vivo* ~8 μm in length, rather widely spaced. Ciliary rows meridional and equidistant; two of them (dorsal brush rows) form dikinetids anteriorly and continue posteriorly as ordinary somatic ciliary rows. Brush rows 1 and 2 composed of 7–11 and 17–25 dikinetids, respectively; row 1 approximately half the length of row 2, both bearing 3–4 μm long immobile, parallel bristles; bristles laying down and possibly covered by epicortical scale layer and hardly recognizable both *in vivo* and in scanning electron micrographs (Figures 4C,E). Brush row 3 extends to the posterior body end, composed of widely spaced monokinetids bearing 2–3 μm long immobile bristles, recognizable both *in vivo* and after protargol impregnation (Figures 4A, 5D,H, 6B,C,D; Table 1). In a few specimens, the row right of brush row 3 commences with shortened cilia as long as brush bristles and continues posteriorly as ordinary somatic ciliary rows.

Oral bulge conspicuous *in vivo* because of extrusomes, 4 × 5 μm after protargol impregnation; cylindrical and set off from body proper and not covered by lepidosomes. Circumoral kinety at the base of the oral bulge, composed of ~11 dikinetids, each bearing a single cilium and inconspicuous nematodesmata extending posteriorly, forming a pharyngeal basket (Figures 4C,E, 5A,D,G, 6C–E,I).

### Phylogenetic Analyses

**Figure 7:**
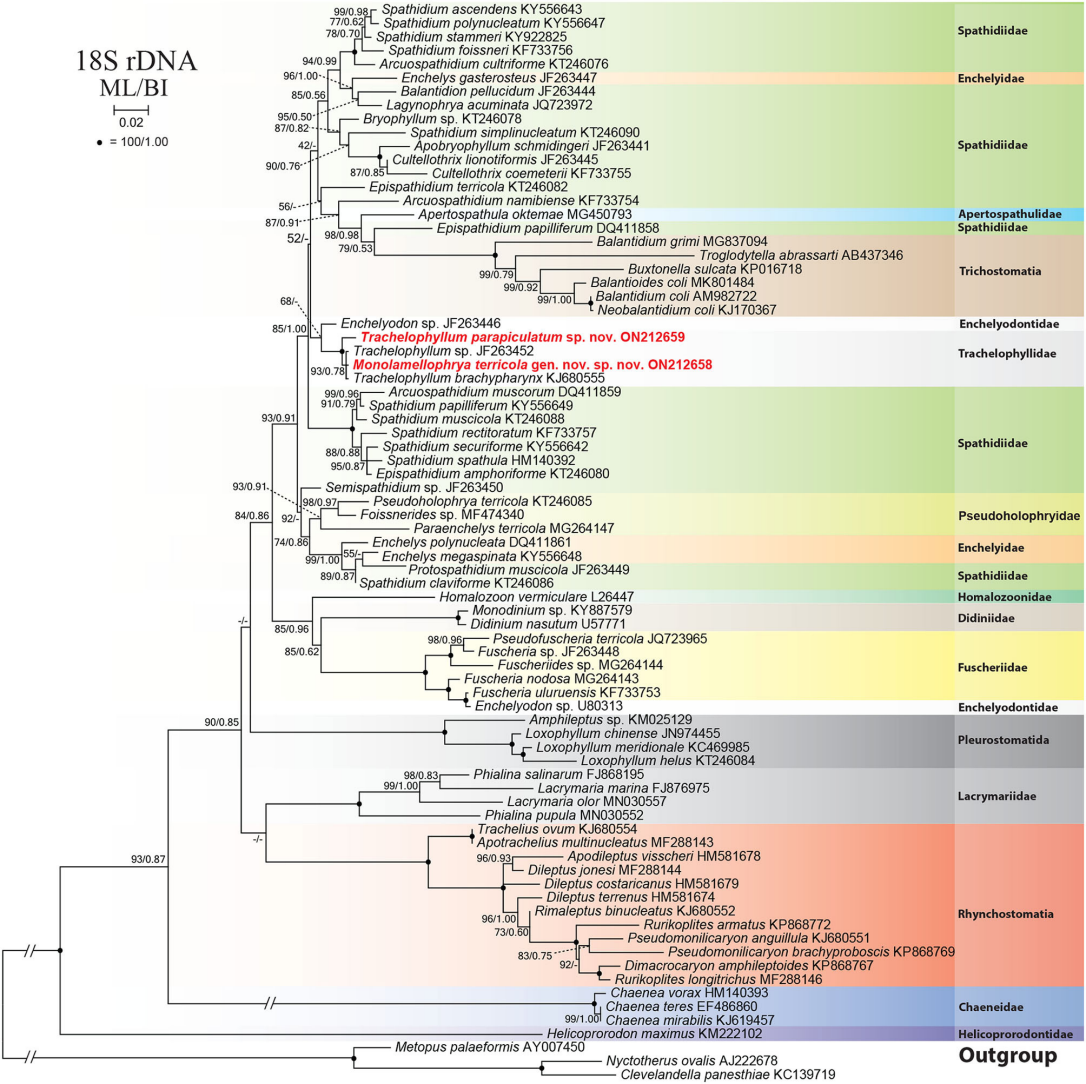
Maximum likelihood (ML) tree based on 18S rRNA gene sequences, showing the phylogenetic position of *Monolamellophrya terricola* gen. nov., sp. nov. and *Trachelophyllum parapiculatum* sp. nov. Newly obtained sequences are in bold. GenBank accession numbers follow species names. Numbers at the nodes represent the maximum likelihood (ML) bootstrap values and the Bayesian inference (BI) posterior probabilities. Dashes indicate bootstrap values < 50%, posterior probabilities < 0.5, or different topologies in BI and ML phylogenies. The scale bar represents two nucleotide substitutions per 100 nucleotides.

**Figure 8:**
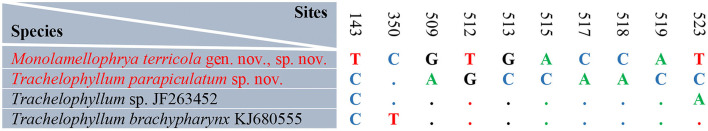
Sequence comparison of the 18S rRNA gene showing the unmatched nucleotides among the new species *Monolamellophrya terricola* gen. nov., sp. nov., *Trachelophyllum parapiculatum* sp. nov., and the other two available trachelophyllid species.

The 18S rRNA gene sequences of *M. terricola* gen. nov., sp. nov. and *T. parapiculatum* sp. nov. are 1,490 base pairs long each, having a GC content of 40.15% and 40.27%, and are available under GenBank accession numbers ON212658 and ON212659, respectively. The phylogenetic trees using ML and BI analyses show rather similar topologies, thus only the ML tree was used (Figure 7). In the phylogenetic tree, the trachelophyllid sequences form a monophyletic clade with full supports. The sequence of *M. terricola* gen. nov., sp. nov. exhibits a similarity of 99.86% (2 nucleotides difference) with both *T. brachypharynx* and an unidentified *Trachelophyllum* sp., and clusters with both sequences with high (93% ML) and low (0.78 BI) support forming a polytomy (Figures 7, 8; Table 2). The sequence of *T. parapiculatum* sp. nov. is placed as a sister branch to these three sequences with full support and shows a similarity of 99.39% (9 nucleotides difference) with both *M. terricola* gen. nov., sp. nov. and *T. brachypharynx* and shows a similarity of 99.46% (8 nucleotides difference) with *Trachelophyllum* sp. (Figure 8; Table 2).

**Table 2:** Interspecific sequence similarity (above diagonal) and number of nucleotide differences (below diagonal) of the 18S rRNA gene sequences among members of the family Trachelophyllidae.

**Species**	**1**	**2**	**3**	**4**
*M. terricola* gen. nov., sp. nov.	-	99.39%	99.86%	99.86%
*T. parapiculatum* sp. nov.	9	-	99.39%	99.46%
*T. brachypharynx* KJ680555	2	9	-	99.86%
*Trachelophyllum* sp. JF263452	2	8	2	-

## Discussion

### *Monolamellophrya* gen. nov. as a New Genus

Members of the family Trachelophyllidae are characterized by the two dikinetidal and one monokinetidal dorsal brush rows and the presence of lepidosomes. Currently, 16 well-characterized trachelophyllid species belonging to eight genera are assigned to the family, mainly based on the types of the lepidosomes (Nicholls and Lynn, 1984; Foissner, 1994, 2005, 2016; Foissner et al., 2002; Bourland, 2017). Only two genera have a single type of lepidosomes each, namely, *Epitholiolus* with only type III lepidosomes and *Trachelophyllum* with only type I lepidosomes. Each of the other six genera has two or three types of lepidosomes. The new genus, *Monolamellophrya* gen. nov., is unique in having only one layer of type II lepidosomes. According to Foissner (2005, 2016) and Foissner et al. (2002), type II lepidosomes have four rather different shapes, and all of them are characterized basically by the cone-shaped superstructure and are found only in the genera *Bilamellophrya* and *Luporinophrys*. The first shape is found only in *Bilamellophrya australiensis* Foissner et al., 2002 and is characterized by the presence of ~10 concave, unevenly spaced arcs rising from the baseplate and fusing together at their ends forming a cone and connected to each other by three transverse rings. The second shape is found in *B. hawaiiensis* Foissner et al., 2002 and *B. fraterculus* Foissner, 2016 and is characterized by the presence of 12–16 scattered polygons in the cone-shaped superstructure. The third shape is found only in *Luporinophrys micelae* Foissner, 2005, and it has a larger size than other type II lepidosomes (4 × 4 μm) and its superstructure is formed by ~20 fine arcs (fibers) in two to five groups rising from the baseplate and fusing together at their ends forming a narrow, curved dome. The fourth shape is simple and consists of approximately seven arcs rising from the baseplate and fusing together forming a cone-shaped superstructure and found in *B. etoschensis* Foissner et al., 2002. The lepidosomes of *M. terricola* gen. nov., sp. nov. are similar to the fourth shape of type II in the shape of the superstructure but slightly differ in the thickness of the arcs (very thin vs. thick) and the shape of the baseplate (slightly to distinctly convex in middle portion vs. obconical). However, the convexity in the middle portion of the baseplate resembles the obconical baseplate described by Foissner et al. (2002) but is more shallow probably due to the preparation procedure. However, this variability in lepidosomes, especially type II, suggests that the trachelophyllids are an underestimated group and more studies are needed to reveal its true diversity.

### Comparison of *Monolamellophrya terricola* gen. nov., sp. nov. With Closely Related Species

*In vivo, M. terricola* gen. nov., sp. nov. is hardly distinguishable from most trachelophyllid species because they have similar appearances and their sizes usually overlap. In addition, the mucilaginous layer of *M. terricola* gen. nov., sp. nov. is not recognizable *in vivo* and after protargol impregnation and thus could be misidentified as a non-trachelophyllid (without mucilaginous layer) haptorid. After protargol impregnation, many trachelophyllid species look similar and even have a similar number of ciliary rows and nuclear apparatus patterns. Thus, the scanning electron microscope is indispensable for the proper identification of such taxa. *M. terricola* gen. nov., sp. nov. is very similar to three *Bilamellophrya* species, namely, *B. etoschensis, B. fraterculus*, and *B. hawaiiensis* especially in body size and shape, the number of somatic kineties, and the overlapping numbers of dikinetids in dorsal brush rows (Table 3). However, *M. terricola* differs from these species mainly by the types of lepidosomes (one type vs. two types). Moreover, *M. terricola* gen. nov., sp. nov. is similar to *B. hawaiiensis* in having acicular extrusomes, but *B. hawaiiensis* has an extra type of extrusomes (rod-shaped). In addition, *B. etoschensis* and *B. fraterculus* each has a different type of extrusome, rod-shaped and oblong, respectively (Table 3) (Foissner et al., 2002; Foissner, 2016).

**Table 3:** Comparison between *Monolamellophrya terricola* gen. nov., sp. nov. and similar species.

**Characters**	***Monolamellophrya terricola* gen. nov., sp. nov**.	***Bilamellophrya australiensis***	***B. etoschensis***	***B. hawaiiensis***	***B. fraterculus***
Size	100–160 × 15–25	150–250 × 25–40	90–150 × 10–20	120–170 × 20–30	130–180 × 15–25
Lepidosomes, types	II	I and II	I and II	I and II	I and II
Extrusomes, types	1	2	1	2	1
Extrusomes, length	7–9	15–20 and 2	4	12 and 2	1
Extrusomes, shape	Acicular	Rod-shape	Rod-shape	Acicular and rod-shape	Oblong
Ciliary rows, number	11–13	22–27	10–12	12–14	12–14
Dikinetids in brush row 1, number	11–18	5–21	11–18	6–18	18–25
Dikinetids in brush row 2, number	12–18	13–26	12–20	10–25	17–29
Reference	Present study	Foissner et al., 2002	Foissner et al., 2002	Foissner et al., 2002	Foissner, 2016

### Comparison of *Trachelophyllum parapiculatum* sp. nov. With Closely Related Species

The trachelophyllid genus *Trachelophyllum* is characterized by having a mucilaginous layer composed only of type I lepidosomes (Foissner et al., 2002; Foissner, 2016). At present, ~31 species are assigned to the genus, most of which probably belong to different litostomatean families (Stokes, 1888; Levander, 1894; Penard, 1922; Dumas, 1930, 1937; Kahl, 1930; Gajewskaja, 1933; Lepsi, 1960; Tucolesco, 1962; Grolière, 1977; Foissner, 1983, 1984; Foissner et al., 2002). However, the lepidosomes of only six species, namely, *T. apiculatum, T. africanum* Foissner et al., 2002, *T. costaricanum* Foissner et al., 2002, *T. lineare* Lepsi, 1960, *T. pannonicum* Foissner et al., 2002, and *T. tachyblastum* Stokes, 1884, are characterized using the scanning electron microscope, and thus their assignment is doubtless. When comparing *T. parapiculatum* sp. nov. with other congeners, we recognized that the genus *Trachelophyllum* is divided into two groups based on the length of dorsal brush row 3: the first group consists of species with bipolar (extends to or near to posterior body end) brush row 3, namely, two populations of *T. apiculatum, T. costaricanum, T. hyalinum* Foissner, 1983, *T. pannonicum, T. clavatum* Stokes, 1886, and *T. parapiculatum* sp. nov.; and the second group consists of all other species and populations either with dorsal brush row 3 similar or slightly longer than row 2 or with brush row 3 of unknown morphology but with special morphological characters such as the coloration, the body shape, or the nuclear apparatus (Foissner et al., 1995, 2002; Foissner, 2016).

Several populations were described as *T. apiculatum*, but they differed from each other by at least a single important character (Table 4), suggesting that they should be classified as distinct species. The Venezuelan neotype population of *T. apiculatum* (population 8 in Table 4) designated by Foissner et al. (2002) has a short dorsal brush row 3, a character that was underestimated by the authors at that time, and differs also from *T. parapiculatum* sp. nov. by the number of dikinetids in dorsal brush row 1 (10–24 vs. 7–11). *Trachelophyllum parapiculatum* sp. nov. could be confused with two different populations described as *T. apiculatum* with long brush row 3: (1) the population described by Foissner (1983) (population 4 in Table 4) differs from *T. parapiculatum* sp. nov. in the extrusomes length (13 vs. 15–24 μm) and the number of dikinetids in dorsal brush row 2 (~8 vs. 17–25); and (2) population II in Foissner (1984) (population 6 in Table 4), which is distinguishable from *T. parapiculatum* sp. nov. by the number of somatic ciliary rows (22–25 vs. 9–13) and the number of dikinetids in dorsal brush row 1 (16–26 vs. 7–11) (Table 4). *Trachelophyllum parapiculatum* sp. nov. is also morphologically very similar to *T. costaricanum* and *T. pannonicum* and can be separated from each of them mainly by the types and shapes of the extrusomes, i.e., *T. parapiculatum* sp. nov. has only one type of rod-shaped extrusomes, while *T. costaricanum* and *T. pannonicum* each has two types of acicular and rod-shaped extrusomes. *T. hyalinum*, which was studied only *in vivo* and after silver impregnation and without morphometric data, differs from *T. parapiculatum* sp. nov. in having longer extrusomes (~30 μm vs. 15–24 μm long) that do not extend into the oral bulge. *T. clavatum* is also characterized by long brush row 3 (i.e., extends to two-thirds of the body length) and differs from other congeners by the presence of a single macronuclear nodule (Stokes, 1888; Foissner, 1983; Foissner et al., 2002).

**Table 4:** Comparison between *Trachelophyllum parapiculatum* sp. nov. and other congeners.

**Characters**		**Size**	**Extrusomes, types**	**Extrusomes, shapes**	**Extrusomes, length**	**Ciliary rows, number**	**Dikinetids in brush row 1, number**	**Dikinetids in brush row 2, number**	**Brush row 3, length**	**Habitat**	**Reference**
*T. parapiculatum* sp. nov.		140–200 × 15–25	1	Rod-shaped	15–24	9–13	7–11	17–25	Long^b^	Freshwater	Present study
*T. apiculatum*	Pop. 1	125–280	1	Rod-shaped	~40	~10	-	-	Short^a^, ^c^	Freshwater	Penard, 1922
	Pop. 2	200	1	Rod-shaped	~20	16–20	-	-	Short^a^, ^c^	Freshwater	Dragesco, 1966
	Pop. 3	130–150	1	Rod-shaped	~20	16–18	-	-	Short^a^, ^c^	Brackish water	Czapik and Jordan, 1976
	Pop. 4	90–110	1	Rod-shaped	13	8–11	8^a^	8^a^	Long^b^	Freshwater	Foissner, 1983
	Pop. 5	120–150	1	Rod-shaped	14	11–12	10^a^	16^a^	Short^a^, ^c^	Soil	Foissner, 1984, pop. I
	Pop. 6	100–170	1	Rod-shaped	21	22–25	16–26^a^	17–25^a^	Long^b^	Freshwater	Foissner, 1984, pop. II
	Pop. 7	50–80	1	Rod-shaped	12–18	19–22	16^a^	16^a^	-	Soil	Song, 1994
	Pop. 8	130–200	1	Rod-shaped	10–20	12–16	10–24	17–26	Short^a^, ^c^	Soil	Foissner et al., 2002
*T. africanum*		150–250 × 20–20	1	Lanceolate	3–4	12–15	9–14	10–22	Short^a^, ^c^	Soil	Foissner et al., 2002
*T. pannonicum*		140–250 × 18–25	2	Acicular and rod-shaped	8–10 and 2	10–14	6–9	12–16	Long^b^	Saline soil	Foissner et al., 2002
*T. costaricanum*		140–220 × 13–20	2	Acicular and rod-shaped	12 and 2	8–10	6–9	13–18	Long^b^	Soil	Foissner et al., 2002
*T. tachyblastum*		90–140 x 10–25	2	Rod-shaped and oblong	12–16 and 2	10–13	4–7	6–15	Short^a^, ^c^	Soil	Foissner, 2016
*T. lineare*		40–350	-	-	-	30	-	-	-	Freshwater	Nicholls and Lynn, 1984

a *From drawings.*

b *Bipolar dorsal brush row 3.*

c *Dorsal brush row 3 as long as or slightly longer than brush 2*.

*Trachelophyllum tachyblastum* resembles *T. parapiculatum* sp. nov. in body shape and size but differs mainly in the length of dorsal brush row 3 (short vs. bipolar), the types and shapes of the extrusomes (two types of rod-shaped and oblong vs. one type of rod-shaped extrusomes), and the numbers of dikinetids in brush rows 1 and 2 (4–7 and 6–15 vs. 7–11 and 17–25, respectively). *T. lineare*, which was described as *Lepidotrachelophyllum fornicis* by Nicholls and Lynn (1984) and Lynn and Nicholls (1985) and as *L. lineare* by Foissner (1994) and Foissner et al. (1999), has a large body size [up to 600 μm according to Lepsi (1960) and 40–350 μm according to Nicholls and Lynn (1984)] and a higher number of somatic ciliary rows (Table 4). *T. brachypharynx* Levander, 1894, which was studied recently by Jang et al. (2015), is a large species (330–445 × 35–45 μm) with filiform extrusomes ~30 μm long and 20–25 somatic ciliary rows. However, the lepidosomes of *T. brachypharynx* were observed only *in vivo* and described as hat-shaped. Thus, the assignment to the genus *Trachelophyllum* is questionable, as noted by Jang et al. (2015).

### Phylogenetic Analyses

The new phylogenetic tree shows that the family Trachelophyllidae is monophyletic. The trachelophyllid clade comprises only four sequences: *M. terricola* gen. nov., sp. nov., *T. parapiculatum* sp. nov., *T. brachypharynx*, and an unidentified *Trachelophyllum* sp. However, like the unidentified *Trachelophyllum* sp., the assignment of *T. brachypharynx* is doubtful because the shape of the lepidosomes was not studied using a scanning electron microscope. According to Jang et al. (2015), the lepidosomes of *T. brachypharynx in vivo* look very similar to type V lepidosomes with their hat-shaped structure as described by Foissner (2005) and, up to date, found only in the genus *Sleighophrys*. Furthermore, the new phylogenetic tree shows a close relationship between the families Trachelophyllidae and Enchelyodontidae (Foissner et al., 2002) based on morphological similarities as discussed by Jang et al. (2015). Clearly, the two families are underrepresented in the phylogenetic tree and obtaining more trachelophyllid and enchelyodontid sequences would be of great help, especially from properly identified species (Foissner et al., 2002; Foissner, 2005, 2016; Jang et al., 2015; Bourland, 2017).

## Data Availability Statement

The datasets presented in this study can be found in online repositories. The names of the repository/repositories and accession number(s) can be found below: https://www.ncbi.nlm.nih.gov/genbank/ [GenBank accession numbers: ON212658 and ON212659].

## Author Contributions

AO and J-HJ designed the study and revised the manuscript. AO performed morphological experiments, molecular experiments, data analyses, and wrote the manuscript. JHM collected the samples and helped in the scanning electron microscopy. All authors read and approved the final version of the manuscript.

## Funding

This study was supported by a grant from the Nakdonggang National Institute of Biological Resources (NNIBR), funded by the Ministry of Environment (MOE) of the Republic of Korea (NNIBR202201105).

## Conflict of Interest

The authors declare that the research was conducted in the absence of any commercial or financial relationships that could be construed as a potential conflict of interest.

## Publisher's Note

All claims expressed in this article are solely those of the authors and do not necessarily represent those of their affiliated organizations, or those of the publisher, the editors and the reviewers. Any product that may be evaluated in this article, or claim that may be made by its manufacturer, is not guaranteed or endorsed by the publisher.
